# Behavior, nutrition and lifestyle in a comprehensive health and disease paradigm: skills and knowledge for a predictive, preventive and personalized medicine

**DOI:** 10.1007/s13167-012-0141-2

**Published:** 2012-03-22

**Authors:** Guglielmo M Trovato

**Affiliations:** 1 Department of Internal Medicine - Diagnostic and Therapeutical Unit, University of Catania, Catania, Italy

**Keywords:** Mediterranean diet, Physical exercise, Health psychology, E-learning, Medical ultrasound, Cluster of medical skills

## Abstract

Health and disease of individuals and of populations are the result of three groups of risk factors: genetics, environment and behavior. Assessment, interventions and tailored changes are possible with integrated approaches more effective if respectful of individuals and different cultures. Assessment tools and integrated interventional strategies are available, but widespread knowledge, skills and competence of well trained individual Medical Doctors still lack. Mediterranean diet is an appropriate reference paradigm because encompasses consistent research background, affordable sustainability, widespread comprehensibility and attractiveness inside a cultural framework of competences and skills in which the Medical Doctors can personally manage the need of prediction (early diagnosis), prevention (intervention on healthy persons) and tailored therapy and follow-up for patients. This profile is flexible and adjustable according to specific needs and preferences due to different economic and ethno-cultural milieus. It can enhanced through on-site/e-learning Continuous Medical Education (CME), by training and using friendly and affordable equipments.

## Introduction

Health and disease of individuals and of populations are the result of three groups of factors: genetics, environment and behavior. Only the last is mostly dependent by the chooses of the single person, but assessment, interventions and tailored changes are possible.

Medical genetics and genetic variation studies relate to human health and disease: knowledge and skills in DNA sequencing and genomics make possible to study the molecular sequences associated with many human diseases, to predict and council accordingly, to prevent and/or treat several conditions. Nutrigenomics is the study of the effects of foods and food constituents on gene expression, and has been associated with the idea of personalized nutrition based on genotype. Nonetheless the actual determinants of human diet are economic, climatic, geographic, historical, with a strong psychological and sociological basis. This is the reason that data on habits, attractiveness of diet, quality of food and alimentation are the more consistent source of knowledge explored by epidemiological tools.

A cultural framework of competences and skills by which Medical Doctors can personally manage the need of prediction (early diagnosis), prevention (intervention on healthy persons) and tailored therapy and follow-up for patients is the premise for reasonable guidelines aimed at more effective clinical practice and favorable cost-benefit balance. Despite the current perception of the usefulness of interventions, also at the clinical individual level, on lifestyles, nutrition and physical activity, and the bulk of studies available, an effective translational practice from the EBM of the epidemiology to the EBM of therapeutic interventions is limited if not totally lacking. Nutritional knowledge and skills teaching and training are a neglected subject of the Curricula of all Schools of Medicine and of allied Health professions. Moreover, we are far from the implementation of skills for enhancing healthy lifestyles, including appropriate physical activity prescribing. This is due to several factors and first of all to excessive focus and expectations related to pharmaceutical approach and results. Moreover, to rely to multiple specialty consultations and procedures for reaching a diagnosis and for managing by follow-up patients is a major disadvantage in terms of time, financial expenses and difficult clinical synthesis and decisions.

The medical doctor can be trained not only, as currently done, to become an improbable medical-financial manager but, as intuitive for anybody, to manage the psychological and nutritional assessment of patients, to extend the power of physical examination by non-invasive, quick and almost inexpensive non-invasive procedures (ECG, ultrasound), and to prescribe what needed, included drugs or other therapies within the scheme of a reasonable follow-up strategy.

## Lifestyles

Lifestyle is a term to describe the way individuals, family circles, and societies live and which behavior they manifest in coping with their physical, psychological, social, and economic environments on a day-to-day basis. It is closely related with the concept of risk [1], with multiple and complex interferences. Substantial proportions of global disease burden are attributable to major risks, to an extent greater than previously estimated. Developing countries suffer most or all of the burden due to many of the leading risks. Strategies that target these known risks can provide substantial and still underestimated public-health gains.

Lifestyle is expressed by daily work and leisure profiles, including activities, attitudes, interests, opinions, values, and allocation of income [2]. From a psychological point of view lifestyle derives from people's self image or self concept (the way they see themselves and believe they are seen by the others), including self-esteem and self-efficacy. Lifestyle is a composite of motivations, needs, and wants and is influenced by factors such as culture, family, reference groups, and social class.

Lifestyle diseases are diseases that appear to increase in frequency as countries become more industrialized and people live longer. Lifestyle diseases share risk factors similar to prolonged exposure to three main modifiable lifestyle behaviors--smoking, unhealthy diet (including alcoholics abuse), and physical inactivity--and result in the development of non-communicable and chronic diseases, substantially degenerative diseases group (heart disease, stroke, diabetes, obesity, metabolic syndrome, chronic obstructive pulmonary disease, and some types of cancer), that can actually be considered consequence of "contagious" behaviors. These conditions imply loss of independence, years of disability, or death, and impose a considerable economic burden on health services. However, despite the well known benefits of a healthy lifestyle, only part of adults follow changes toward healthier lifestyles, and usually only for some of them; nonetheless, through other factors, also pharmacological, prevalence is declining and development and consequence of unhealthy lifestyles delayed. This approach, i.e. tardy interventions, is certainly expensive, and not easily affordable by many societies and individuals. The challenge of promoting physical activity is as much the responsibility of governments, as of the people. However, individual action for physical activity is influenced by the environment, sports and recreational facilities, and national policy. It requires coordination among many sectors, such as health, sports, education and culture policy, media and information, transport and mobility development, urban planning, local governments, and financial and economic planning.

**Figure 1:**
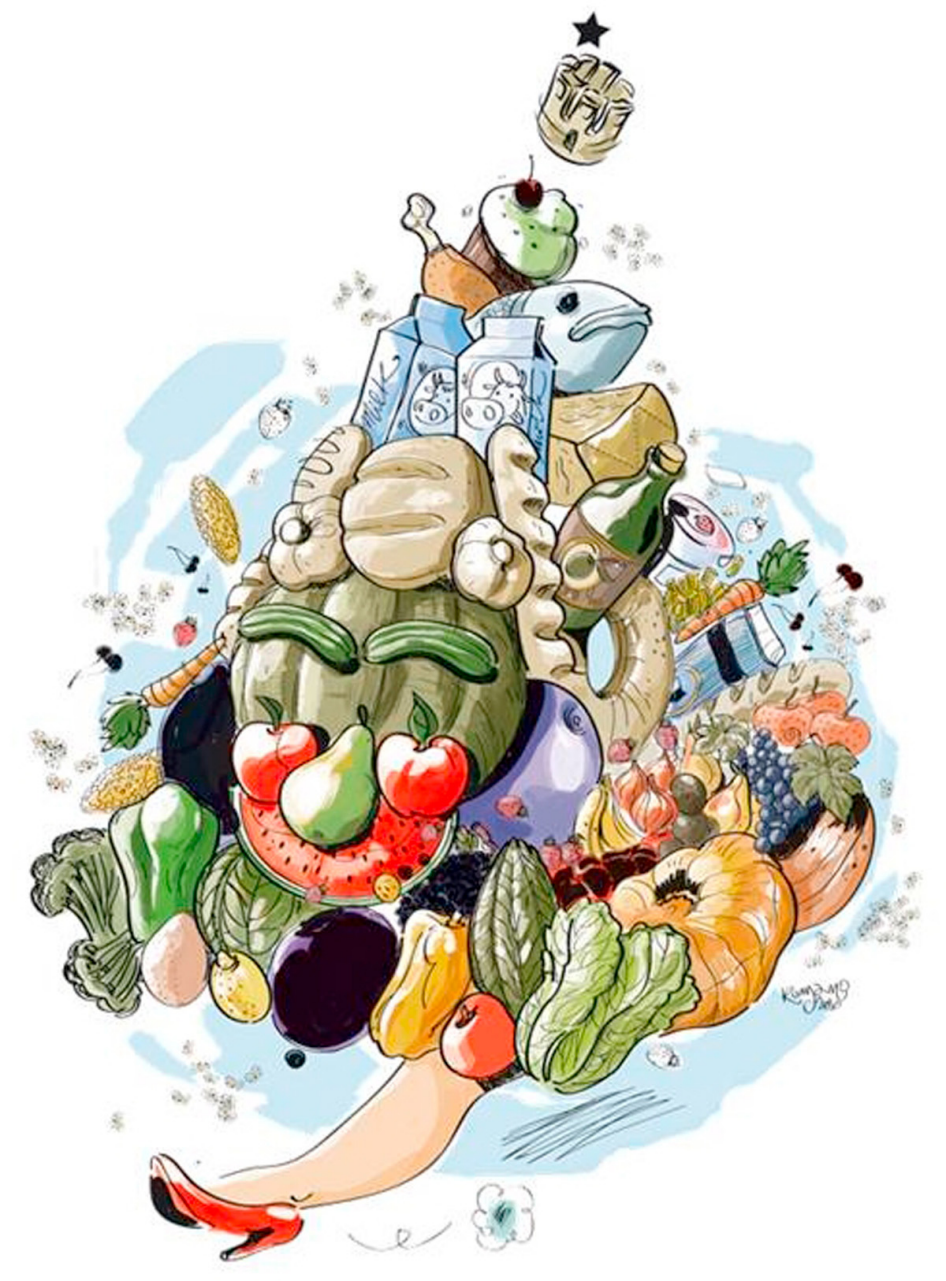
**Dietary/physical exercise Arcimboldo, which visually depicts guidelines and ideas is expressly designed by Giuliano Cangiano, by courtesy**. It represents the preferred food, according to current guidelines, from those to be most frequently chosen to those to be exceptionally included. The Arcimboldo is running, obviously: practicing physical exercise.

The combination of the main healthy lifestyle factors--maintaining a healthy weight, exercising regularly, following a healthy diet, and not smoking--seem to be associated with as much as an 80% reduction in the risk of developing the most common and deadly chronic diseases. This reinforces the current public health recommendations for the observance of healthy lifestyle habits, and because the roots of these habits often originate during the formative stages of life, it is especially important to start early in teaching important lessons concerning healthy living. The complex puzzle can appear like a Arcimboldo's drawing, in which many components are integrated into an unique figure (Figure 1). The first recent evidence that lifestyle intervention are powerful and beneficial also on the actual levels of disease biomarkers [3], including the traditional cardiac risk factors and emerging biomarkers, is a further reason for following this way.

## From the risk factors to the PPPM

The medical study of lifestyles, since the first ones of more than 50 years ago [4,5] is actually coincident with and derived by the study of risk factors for several disease [6,7]: the primary goals were using them as predictive tools, helpful for life insurances and for physicians and health institutions interested in prevention. The subsequent approaches were aimed at the translation of these information to clinical individual strategies.

The advent of personalized medicine [8,9] as a medical model emphasizing the customization of healthcare, with all decisions and practices being tailored to individual patients in whatever ways possible, is changing this scenario. The current development of stratified medicine, i.e. the management of a group of patients with shared biological char-acteristics [10], is now possible and is bringing a further advance to medicine.

Molecular diagnostic testing, molecular profiling technologies, including proteomic profiling, metabolomic analysis, genetic testing and nutrigenomics, are beginning to be used to select the best therapy in order to achieve the best possible medical outcome for that person or group [11,12].

Medicine and human civilization, share the same history of people aimed at an own personalized care, in health and disease. The goals are to prevent pain and/or illnesses, to predict what could be beneficial and what could be detrimental and to act appropriately with an adequate attention to persons.

The oldest approaches were based on observations and experience but also economical, political and religious needs and beliefs were and are strongly operating [13]. This "heritage", the tradition of medicine, which had to be adapted to different environment and, namely, climate, is now preserved, modified or decidedly revolutionized by more scientific approaches [14]: these are currently included inside the definition of evidence-based medicine (EBM).

The steps of the recent history of predictive, preventive and personalized medicine (PPPM) can be summarized as follows:

1) The definition of risk factors or determinants of health and disease derived from extensive, prolonged and still now ongoing epidemiological studies (Framingham, Seven Countries Studies and, thereafter, many others, most recent and elsewhere) that discovered the most critical *predictive indexes of disease*.

The corresponding side of this approach was and is the search for biological markers (genetic, biochemical, derived by imaging procedures) to be used individually or in more integrated algorithms.

2) The *preventive approach *through population or worldwide interventions, and by smaller risk groups intervention, driven by institutional guidelines and definite laws and rules. In this case the biomarkers are used as monitoring tools.

3) The *personalized approach *is the clinical side of a professionally EBM. It includes contributions from advanced molecular and imaging diagnostics, innovative histopathology definitions, and pharmacology. The neglected skills and competence are implicit and even more valuable. They are in the domain of healthy lifestyle, and encompass strategies for enhancing quality of nutritional profiles, physical activity patterns, and socio-environmental conditions. These last can be positively modified also at the individual level (noise, neighbours, accessibility and even minor architectural barriers).

The sustainability of this approach can appear doubtful, if not impossible outside highly organized and advanced health Institutions. This is not true. The core subject of our integrated clinical work (MD, psychologist, dietician and sport and exercise medicine MD specialist), is developed in our as in other Internal Medicine Unit along these lines, with easily affordable integrations when necessary.

This methodology becomes affordable when a consistent and reliable clustering of diagnostic skills is present in a circle of few specialists; they must be focused at the early non-invasive diagnosis, to be refined with more advanced procedure when necessary, and at the appropriate and timely accomplishment of pharmacological, nutritional, behavioral and exercise therapeutic interventions. With an adequate but currently widespread and up-to-date medical-scientific background, with the support of a knowledge networks also e-learning based, a substantially sustainable approach to PPPM is possible everywhere.

It is noteworthy that greater epidemiological studies, even attempting to focus at the effects of lifestyle changes [15], are able to give quite generic information based mostly on minor changes defined by averages that become significant as a consequence of the numerousness of the studied population and groups.

## Risk markers and indexes

A risk marker, also called, specially as a group, risk index, is a variable that is quantitatively associated with a disease or other outcome. This is an epidemiological information, and means that direct alteration of the risk marker does not necessarily alter the risk of that disease or the probability of that outcome. A risk factor, not necessarily coincident with the risk marker, is a condition that actually determines one or more diseases. The modern concept stems from the Framingham Heart Study [16]. This is a long-term (1948-), still now ongoing cardiovascular study on residents of the town of Framingham, Massachusetts. Among the identified risk factors someone is a real disease, like diabetes and hypertension, other are habits (smoking, sedentary life), other are more markers, biochemical (cholesterol, triglycerides, uric acid), or not (left ventricular hypertrophy by ECG, or atrial fibrillation) of a risk or of a disease, not the disease or the risk itself. The outcomes of existing risk factors includes primarily myocardial infarction, sudden death, congestive heart failure, cerebral stroke. The epidemiological study in the subsequent generations of this Framingham population is gaining information on several aspects, including diet and other habits [17]. The concept of risk and causative factors for many disease, specially lung and cancer disease, is inside the mind of people and researchers, and the current state of the knowledge is wide and complex.

## Social determinants of health

These are the economic and social conditions under which people live which determine their health. They are "societal risk conditions", rather than individual risk factors that either increase or decrease the risk for a disease [18,19]. The prediction of adult incidence and death from disease is related to income differences which are also strongly related to the health of children and youth. Worldwide children living in low-income families are more likely to experience greater incidence of a variety of illnesses, hospital stays, accidental injuries, mental health problems, lower school achievement and early drop-out, family violence and child abuse, among others. Social determinants of health are blended throughout the life of individuals and populations to the more traditional and better studied risk factors.

The socioeconomic circumstances of individuals and groups are equally or more important to health status than medical care and personal health behaviors, such as smoking and eating patterns. But depend from complex and not easily modifiable factors. Nonetheless, in the mean-while, we cannot wait only a more global, worldwide or national approach. We have the awareness that financial and intellectual resources in any State or Nation are not always adequate for a timely and even depoliticized approach. So, the search for appropriate strategies aimed at improving individual or group health and, if possible, quality of life, is warranted. The awareness that medical doctors, and all health professionals, with their advices, prescriptions and, why not, personal examples is important. This is the point from where it is possible to move significant steps toward the understanding of the complex and a relevant component of an integrated approach to the management of health determinants. This is a core position of the strategy for working for healthier persons and societies and a key step toward civilization.

The domino effect of the tenacious persistence of medical concepts and the individual effort of their application must be carefully considered and appropriately used. This is confirmed by the interventional studies that changed incidence and prevalence of disease acting on one particular strategy (smoking, alcohol, excessive fats in diets withdrawal). All these have specific and more general effects, even when focused to small population group. Moreover, a lesson is that even interventions on single risk factors can have positive effects for other apparently non related risk factors. Of course, neutral or somewhat unfavorable effects are possible, as in the case of smoking withdrawal and body weight increase [20]: this information warrants a more integrated strategy, including pro-active nutritional and physical activity counseling.

## Sedentary lifestyle

Sedentary lifestyle is a medical term used to denote a type of lifestyle with no or irregular physical activity. The negative connotation is a recent concept, that stems from epidemiological as well from intervention studies [21].

Sedentary activities include sitting, reading, watching television and computer use for much of the day with little or no vigorous physical exercise.

Physical inactivity is linked to almost all common health problems including cardiovascular diseases, type II diabetes, obesity/overweight, cancer, dementia and depression. Furthermore, the great value of physical activity in the prevention and treatment of disease has been proven over recent years. Physical activity is essential for improved health as well as for longevity [22].

Over recent years, 'physical activity on prescription' has proven to be a feasible way to increase an individual's or patient's physical activity levels: It has been suggested that leisure-time activity will be insufficient to prevent increasing population levels of obesity and chronic diseases, and it may be necessary to focus on decreasing sitting and increasing activity in transport and at work to restore the energy balance that resulted in a much-more-stable body weight [23,24].

The promotion of physical activity is not only a matter of health education and of health behavioral strategy but is effective and feasible by the 'physical activity on prescription' as already in use in Denmark, Sweden [25], New Zealand and in few Italian Regions [26].

**Figure 2:**
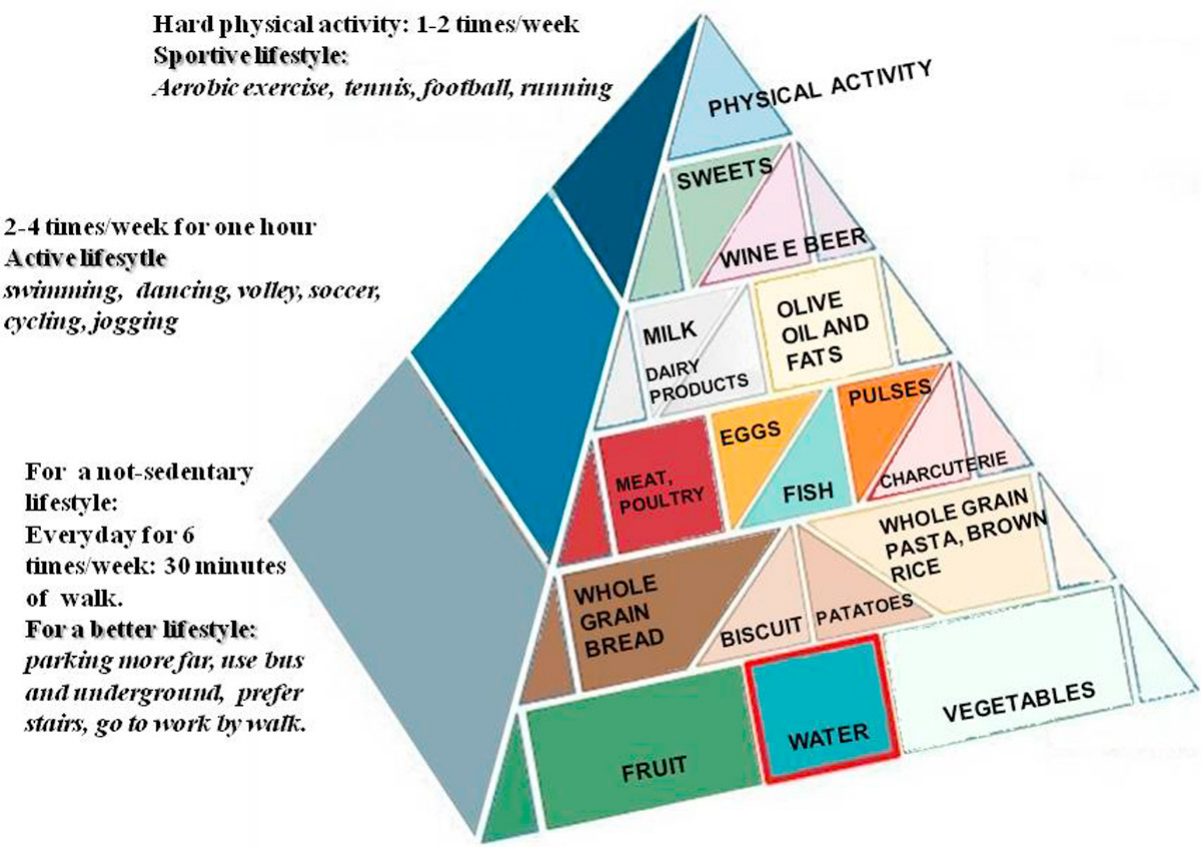
**This pyramid is more focused to the physical activity suggestions merged using the current guidelines and our clinical strategy**. Physical activity is recommended with a daily exercise habit (10.000 steps, no elevators, no cars) and regular aerobic more intensive physical exercise (3 times/week) according to specific needs and possibilities.

A reliable methodology of assessment of physical activity is a core point for any quantitative approach in epidemiology and clinics. One of them, used mainly as a support for nutritional assessment, is the Baecke Questionnaire, that can be managed easily by doctors and health professionals [27] assessing physical activity at work, sport during leisure time and physical activity during leisure time excluding sport. The key recommendation against sedentary habits can be summarized with a pyramid, in which dietary advices are integrated with general physical exercise prescriptions (Figure 2).

## Nutritional profiles - guidelines

Diets and recommendations are continuously provided also as official guidelines by numerous medical and governmental institutions. The attempt and the perspective is the promotion of overall and/or of certain aspects of health. Advances in the fields of molecular biology, biochemistry, and genetics enhanced the study of nutrition that is increasingly concerned with metabolism and metabolic pathways: the sequences of biochemical steps through which substances in living things change from one form to another. The clinical experience and knowledge are contributing to novel dietary recommendations for the general population.

### Institutional driving of healthy diet

Particularly within the last years several government agencies have attempted to combat the amount and method of media coverage plentiful upon "junk" foods. Governments also put pressure on businesses to promote healthful food options, consider limiting the availability of junk food in state-run schools, and tax foods that are high in fat. Vending machines in particular have come under fire as being avenues of entry into schools for junk food promoters. However, there is little in the way of regulation and it is difficult for most people to properly analyze the real merits of a company referring to itself as "healthy." There are different ways to interpret the food-health relationship and make sense of the evidence about healthy eating in the everyday experience. With this premise, the need of a personalized health psychology approach is needed [28].

## Functional food

There is since several years the scientific evidence that some foods and food components have beneficial physiological and psychological effects over and above the provision of the basic nutrients. Moreover, the concept of "positive" or "optimal" nutrition, has its focus more to the quality and adequacy of dietary patterns than to the strategy of eliminating or avoiding "dangerous" or "unhealthy" food. Fruits, vegetables, legumes, olive oil, whole grains, wine and milk have been found to contain components with potential health benefits. On these basis, new foods are being developed to enhance or incorporate these components potentially beneficial for health through evidences derived from epidemiological, clinical and/or pathophysiological studies.

In general, functional food are consumed as part of the normal diet and are considered useful and "functioning" because contain in significant quantity biologically active components which could enhance health or reduce risk of disease. Very similar, in concept, to drugs, it is reasonable to have in mind that excessive amount of a "functional" food can harm as a consequence of the unfavorable effects of its component. The key point is that any food product and its related claims should be placed in the context of diets and analyzed from a bio-psychosocial standpoint.

The communication of health benefits of one or most "functional food" to consumers is of critical importance and the knowledge must be reliable and up-to-date in order to allow informed choices about the foods to eat and enjoy [29].

Many "functional food" are key components of Mediterranean diet, and, at least equally important, also the cooking modalities are usually consistent with the need of preserving the physiologically effective components [30]. This is not the minor reason for supporting a comprehensive approach warranting strategies for healthier dietary profiles by easily and friendly concepts and messages.

There is widespread belief that organic food is significantly safer for consumption than food grown conventionally. But this belief is not systematically based on scientific evidence. Nonetheless, the awareness that foods claiming to be organic are free of artificial food additives, and are often processed with fewer artificial methods, materials and conditions, such as chemical ripening, food irradiation, and genetically modified ingredients is more comfortable for most consumers. Pesticides are allowed so long as they are not synthetic. In this subset also botanically-derived insecticides have gained favor in recent years, due in part to the perception that, because they originate from plant material, they are more safe or "natural" [31,32]. These pesticides are often used for growing crops organically, according to guidelines set forth by certification programs, and may also find favor in organic food production, both in the field and in controlled environments. Valuable contributions to domestic food production are present in countries where strict enforcement of pesticide regulations is impractical.

## Translational research and medicine

Evidence-based medicine (EBM) or evidence-based practice (EBP) applies the best available evidence gained from the scientific method to clinical decision making [33]. It assesses the strength of evidence of the risks and benefits of treatments (including lack of treatment) and of diagnostic tests. Translational medicine, as most EBM, is a medical practice based on interventional epidemiology. It is a natural progression from EBM. It integrates research from the basic sciences, social sciences and political sciences with the aim of optimizing patient care and preventive measures which may extend beyond healthcare services. It implies the process of turning appropriate biological discoveries into drugs and medical devices that can be used in the treatment of patients. Translational research is a paradigm for research alternative to the dichotomy of basic research and applied research [34]. It is often applied in the domain of medicine but has more general applicability as a distinct research approach. It is also allied in practice with the approaches of participative science and participatory action research. As a general concept, it is a cross disciplinary scientific research that is motivated by the need for practical applications that help people. So, the primary goal of "translational" research is to integrate advancements in molecular biology with clinical trials, taking research from the "bench-to-bedside".

Continuous improvements of community-based approaches, and also effective and sustainable approaches for prevention become possible with the epidemiological and biological premise to translation of researches into interventions. Considering health determinants and risk factors, this needs an integrated view of educational and environmental actions to facilitate greater physical activity, together with fiscal and regulatory changes to promote production, promotion, and delivery of healthier meals and total food supply.

Practitioners of any health profession, scientists, economists and food producers and suppliers, media responsible persons, policy makers, and the public need sound evidence from these different and new research methods. These approaches involve both experimental and non-experimental methodologies, but are also sensitive to cultural and ethnic priorities.

There is the need of an universal reference paradigm, easily understandable and communicable, sustainable and with a consistent scientific support, sufficiently comprehensive and attractive in different countries and population, to be promoted in a globalized society, such the world is now.

## Mediterranean diet as an unifying reference paradigm for healthy lifestyles

The history of Mediterranean diet is the same history of culture, populations and economies of the Mediterranean area: by this approach it is very easy to misunderstand its meaning. In the Old Greek Medicine, and it means also Magna Graecia and Sicily, of course, a profile of healthy diet was already present, and was very similar to that one that we consider now "Mediterranean".

The "discovery" of the benefits of Mediterranean diet by the Rockefeller Foundation Missions and by Ancel Keys during and immediately after the Second world war was the natural consequence of the widespread but vague ideas asserting that Mediterranean food and culinary traditions were beneficial to health. The Seven Countries Study is the first study that systematically examined the relationships between lifestyle, diet, coronary heart disease and stroke in different populations and in different regions of the world. It directed attention to the causes of coronary heart disease and stroke, but also showed that an individual's risk can be changed [35]. The Seven Countries Study showed that increased cholesterol (hypercholesterolemia) increases cardiovascular risk both at the population level and at the individual level. It demonstrated that the association between increased cholesterol and coronary heart disease (CHD) is homogeneous across different cultures. In addition, in the subgroup of participants who suffered from cancer, the study revealed that increased cholesterol and being overweight or obese increases mortality from cancer.

The conceptualization of what more exactly is meant by «Mediterranean diet» today, and its benefits, was performed, amongst others, by Walter Willett, Harvard University. Since then contribution from several parts of the USA, Europe and mainly from Greece, Italy and Spain gave a further progress to knowledge. For instance, Mediterranean diet, articulated into extensive lifestyles interventions in a clinical follow-up study, improves renal artery circulation, decreasing renal resistive index, even without significant modifications of insulin resistance. This is a beneficial effect and modifies the pathophysiology of essential hypertension [36]. The effects on autonomic nervous system [37] and on oxidizing processes [38] can be the key factors in the prevention of cardiovascular disease. Also elderly cognitive impairment and Alzheimer disease are conditions associated with a very lower prevalence in subject on Mediterranean diet [39].

## The pyramid of mediterranean diet

**Figure 3:**
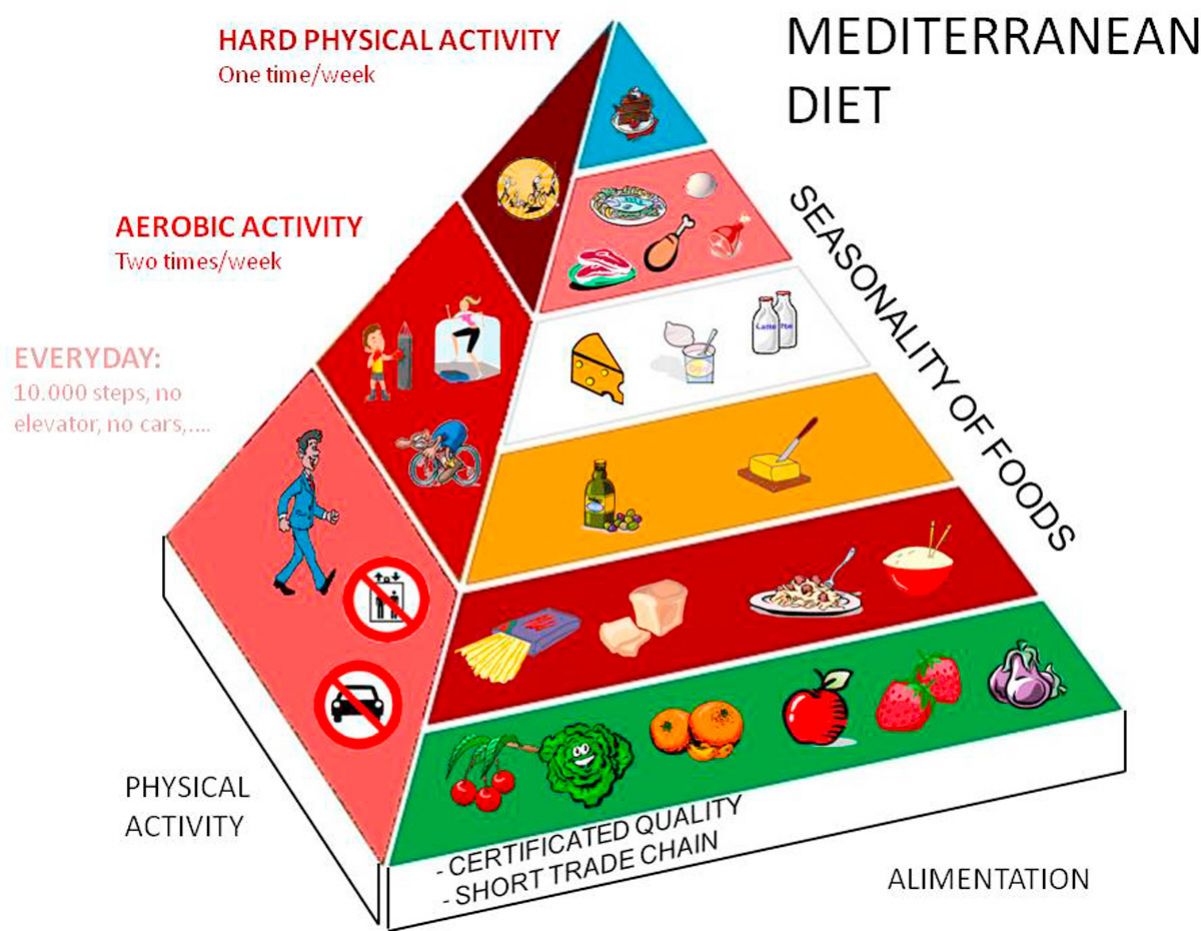
**This pyramid represents both the nutritional and the physical activity suggestions merged using the current guidelines and our clinical strategy**. According to the Harvard-led group a dietary pyramid has been developed to describe the Mediterranean dietary pattern. This pattern consist of: 1) daily consumption of nonrefined cereals and products (e.g., whole-grain bread, pasta, brown rice, and the like), fruits (4 to 6 servings/day), vegetables (2 to 3 servings/day), olive oil (as the main added lipid), and non-fat or low-fat dairy products (1 to 2 servings/day); 2) weekly consumption of fish, poultry, potatoes, olives, pulses, and nuts (4 to 6 servings/week), as well as more rarely eggs and sweets (1 to 3 servings/week), and monthly consumption of red meat and meat products (4 to 5 servings/month). It is also characterized by moderate consumption of wine (1 to 2 wine glasses/day), moderate consumption of fat, and a high monounsaturated to saturated fat ratio. The quality of food is characterized by three main features: 1) short trade chain; 2) certified quality and 3) preference to the use of season's food, particularly fruits and vegetables.

According to the Harvard-led group a dietary pyramid (Figure 3) has been developed to describe the Mediterranean dietary pattern. This pattern consist of: 1) daily consumption of nonrefined cereals and products (e.g., whole-grain bread, pasta, brown rice, and the like), fruits (4 to 6 servings/day), vegetables (2 to 3 servings/day), olive oil (as the main added lipid), and non-fat or low-fat dairy products (1 to 2 servings/day); 2) weekly consumption of fish, poultry, potatoes, olives, pulses, and nuts (4 to 6 servings/week), as well as more rarely eggs and sweets (1 to 3 servings/week), and monthly consumption of red meat and meat products (4 to 5 servings/month).

It is also characterized by moderate consumption of wine (1 to 2 wine glasses/day), moderate consumption of fat, and a high monounsaturated to saturated fat ratio.

The evaluation of the nutritional habits that we currently use since 15 years in our Medical Unit according and updated to Italian Nutritional Guidelines (Dietosystem^®^) is based on a validated food-frequency questionnaire. We ask all participants to report their weekly average intake of several food items that they consumed during the last week, and then trying to extrapolate the average consumption during the last year. The frequency of consumption is quantified in terms of the number of times a week each food group was consumed. Thus a value of 0 was assigned to Mediterranean food items (see below) rarely or never consumed. Alcohol consumption is measured in wine glasses (100 ml) and quantified by ethanol intake (g/drink). One wine glass is equal to 12 g ethanol concentration.

According to the previous dietary pattern and the reported weekly frequency consumption of these food groups, we calculate each participant's diet score, which assessed adherence to the Mediterranean diet (range 0 to 55).

For alcohol the daily base is used, and the greater daily use in the past week is used for scoring. We assign a score of 5 for consumption of 0-2 red wine glasses/day for men, 1 red wine glasses/day for women, and negative (unfavourable) scores for greater consumption of more than 2 red wine glasses/day (1 for women). Other alcoholics use is calculated as a negative value, according to alcohol grams: for 0-10 the score is 0, 10-20 the score is -1, more than 50 g/day the score is -5 (see below).

Higher values of this diet score indicate greater adherence to the Mediterranean diet, whereas lower values indicate adherence to the "Westernized" diet. We recently reported our experience on Mediterranean diet score (range 0-55).

Mediterranean food (pasta and rice; whole-grain bread, brown rice, legumes; fruit; green vegetables; fish, poultry; nonfat or low-fat dairy products; olive oil) had assigned a score of 0: no consumption, a score of 1 for 1 to 4 times/w, 2 for 5 to 8 times/w, 3 for 9 to 11 times/w, 4 for 12 to 14 times/w, and 5 for more than 14 times/w; for westernized food (red meat; dairy products-butter; potatoes and eggs; cakes) opposite scores were assigned (i.e., 0 when a participant reported more than 5 weekly consumption to: 5 for no weekly consumption, score 4 for 1 weekly consumption, 3 for 2 weekly consumption, 2 for 4 weekly consumption, 1 for 5 weekly consumption); wine and alcoholics (0-10 g of alcoholics from red wine for women score 5; 0-20 g of alcoholics from red wine for men score 5; each increment of 10 determines negative scores (20-30 = -1. 30-40 = -2. 40-50 = -3. 50-60 = -4, >60 = -5 for men; 10 less for women and for all non-wine alcoholics,: 10-2 = 0-1; 20-30 = -2; 30-40 = -3; 40-50 = -4; >50 = -5). A tentative, arbitrary cut-off for sufficient adherence to Mediterranean diet can be defined as a score >35.

**Figure 4:**
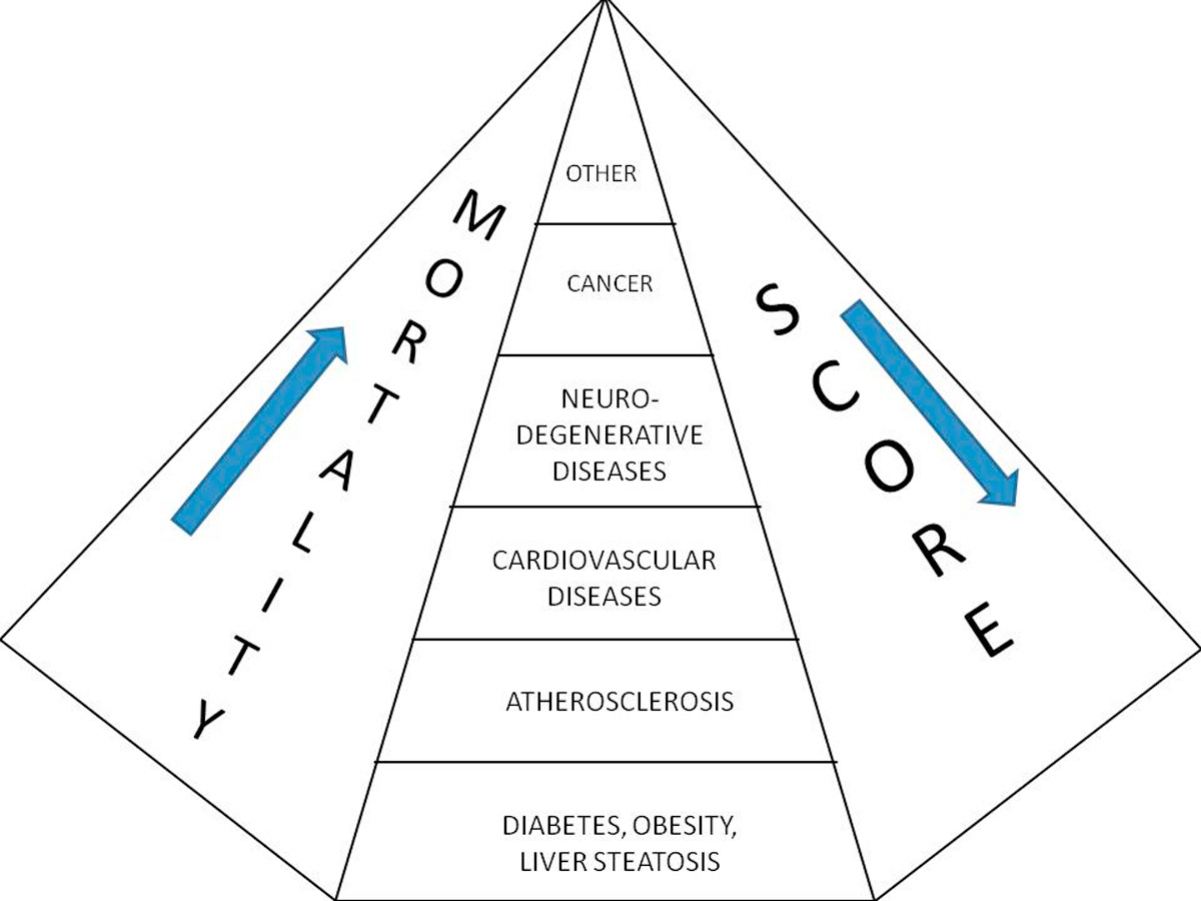
**Mediterranean diet score adherence and mortality**. Mortality and diseases increase along the decrease of the adherence to a Mediterranean diet profile, which is assumed as a proxy to the current European and North American lifestyle-nutritional guidelines.

Mediterranean score was assessed in 8138 healthy non-diabetic, overweight/obese subjects,1996-2010, referred for US liver diagnostic and dietary counseling. In this population there is a trend throughout the last 15 years toward the loss of the adherence to Mediterranean diet from 37.06± 3.213 to 34.82 ± 5.014 p < 0,0001, not associated with BMI or physical activity change (Figure 4).

This information, that can be also interpreted as the loss of adherence to the most internationally recognized guidelines for healthy nutrition, reinforces reasons and need for coordinated and sustainable interventions. There are different possibilities of adapting the main lines of the EBM nutritional recommendation to actual individual and population conditions. Cultural and traditional habits, gender, age, climate, financial and marketing affordability are all conditions that can be optimally managed, using appropriate skills and knowledge along adequate software applications, that can be tailored inside the frame of the current healthy nutrition and lifestyle guidelines.

Non-profit organizations, and particularly Oldways, in USA, along Federal actions (Let's move program and others) gave and are giving significant practical contributions that appear particularly effective because are based on friendly intervention strategies. The same efforts and interventions are not easily recognizable in Europe, even with many limited national and supranational interventions.

## Mediterranean diet: food, recipes and cooking

Mediterranean diet and clinical nutrition are both neglected tools that are useful in the prevention and treatment of cardiovascular diseases. Web- and e-learning course on life-style and Mediterranean diet are, hopefully, a step toward a greater dissemination of these information.

Examples and modern culinary strategies in agreement with Mediterranean diet "best practice" are available: cookery books, web and video recipes, and also institutional, academic courses. Videos are available on the web, but actually the daily work done by dieticians with patients, their relatives and friends using also telephone and social network empowering systems (mainly facebook and, for individual empowering, skype and several windows/Mac applications, also for tablets and smartphones) are innovative important instrument.

The paradigm of Mediterranean diet, among other confusing and not EBM based schemes and profiles, is a clear message addressing choice of food, balance, cooking skills that is connected and articulated with related lifestyles--physical activity and daily rhythms counseling. It can be optimally tailored if the medical doctors have the competences to address their own prescription or, better, the integrated strategies of dieticians and psychologists trained for managing alimentary behavior.

**Figure 5:**
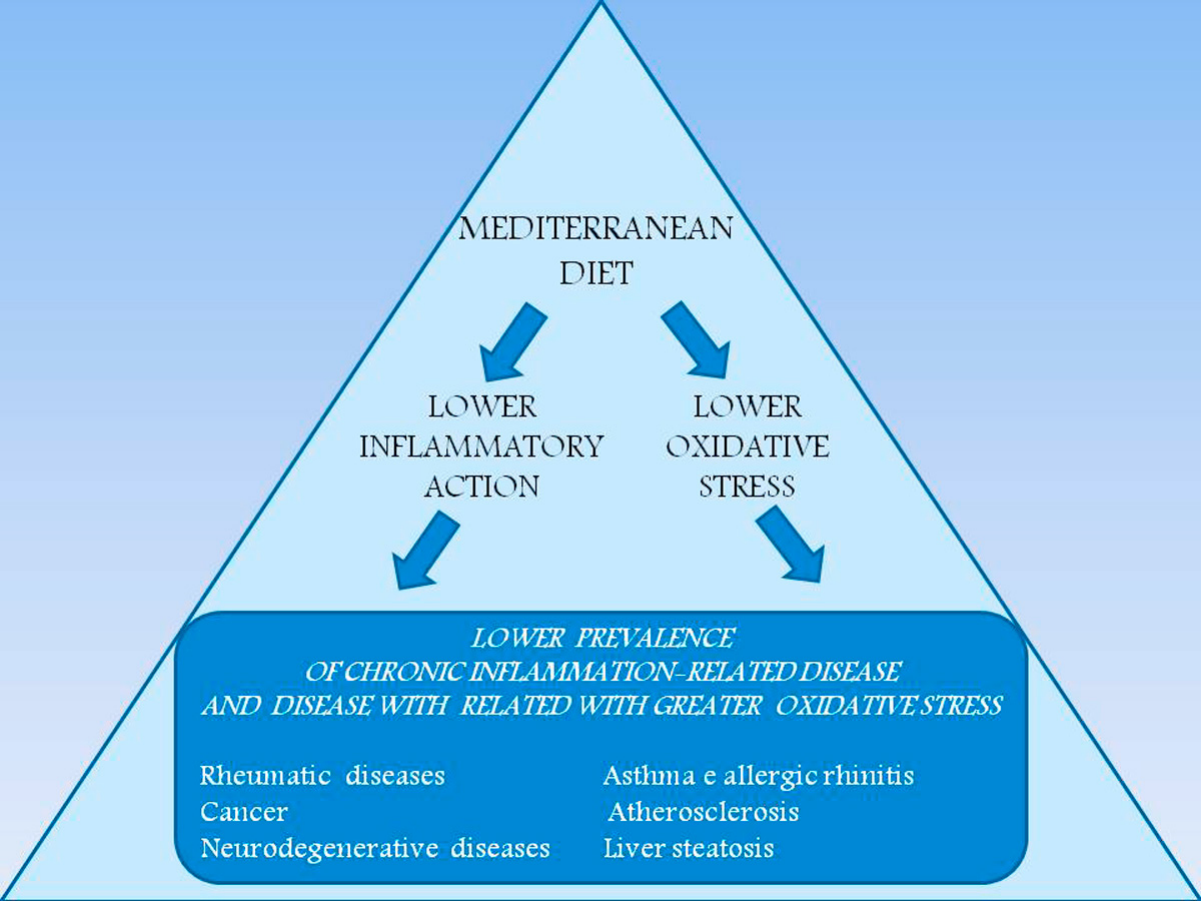
**Anti-inflammatory mechanisms of Mediterranean diet are presented according to EBM studies**. Nutrients' profile of Mediterranean diet, and of the present Italian and USA guidelines is associated with lower inflammatory action and lower oxidative stress of its components. These are the two main features of Mediterranean diet composition which explain the lower prevalence of related disease (rheumatic, allergic, degenerative including atherosclerosis, neurological, metabolic, cancer) in clinical and epidemiological studies. Greater adherence to Mediterranean diet profile is associated with lower prevalence and severity of these conditions.

**Figure 6:**
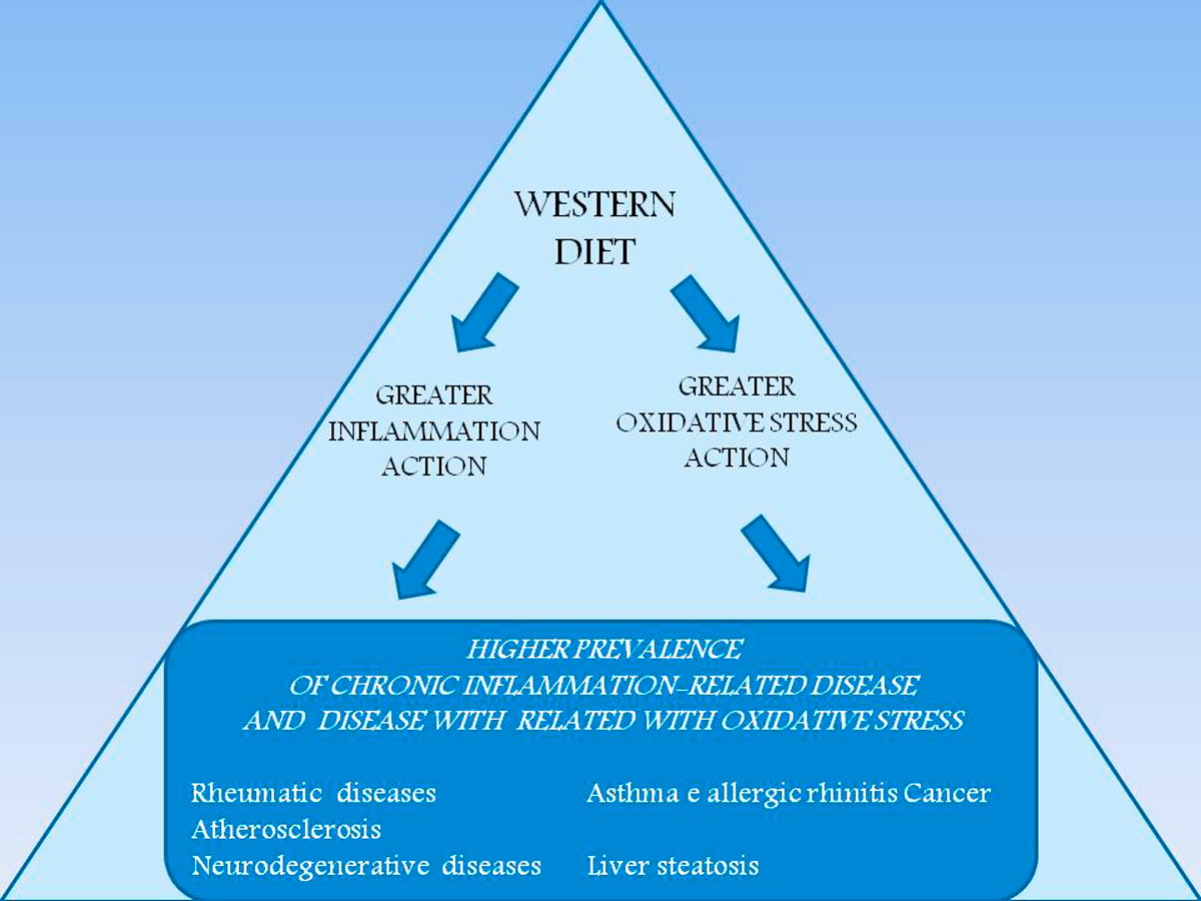
**Pro-inflammatory mechanisms of Western diet are presented according to EBM studies**. Nutrients' profile of western diet is associated with greater inflammatory action and greater oxidative stress of its components. These explain the greater prevalence of related disease (rheumatic, allergic, degenerative including atherosclerosis, neurological, metabolic, cancer) in clinical and epidemiological studies. Lower adherence to Mediterranean diet profile is associated with greater prevalence and severity of these conditions.

There is the confirm, from the intentional modification of dietary profiles assessed by the Alternative Healthy Eating Index (AHEI) in the Whitehall II cohort [40], that changes toward patterns with higher nuts and soy, total fiber, and, to a lesser extent, ratio of white to red meat were associated with a decreased risk of mortality independent of other components. These findings, based on a 18 years lasting study, are consistent with those of several studies that have investigated other diet-quality scores and mortality; these studies support an inverse association between Mediterranean diet score and all-cause, CHD, and cancer mortality. Mediterranean diet score, which measures adherence to a traditional Mediterranean type of diet characterized by high intake of fruit, vegetables, cereal, potatoes, and fish; high ratio of polyunsaturated fatty acids to saturated fatty acids; low intake of meat (white and red) and dairy products; and moderate alcohol consumption. A protective effect of moderate alcohol consumption on all-cause and CVD PMA Journal mortality, independent of other AHEI components was also shown. The putative mechanisms are complex, but mainly include a reduced pro-inflammatory effect of Mediterranean diet (Figure 5) in comparison with a strong unfavorable effect of western diets (Figure 6).

**Figure 7:**
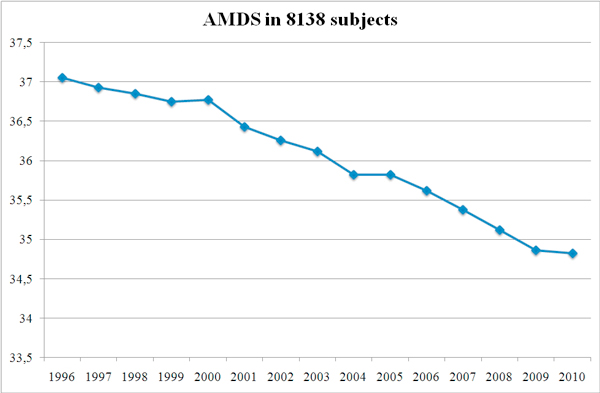
**Adherence to nutritional guidelines shows a continuous decline in the past 15 years**. This is our experience on Mediterranean diet score (range 0-55). It was assessed in 8138 healthy non-diabetic, overweight/obese subjects, 1996-2010, referred for US liver diagnostic and dietary counseling. In our population there is a trend throughout the last 15 years toward the loss of the adherence to Mediterranean diet from 37.06 ± 3.213 to 34.82 ± 5.014 p < 0,0001, not associated with BMI or physical activity change. This information, that can be also more generally interpreted as the loss of adherence to the most internationally recognized guidelines for healthy nutrition, reinforces reasons and need for coordinated and sustainable interventions.

The Whitehall study is the first to provide epidemiologic evidence that enhancing adherence to dietary recommendations of the AHEI may decrease the long-term risk of all-cause and CVD mortality. The WHEL study, in San Diego, demonstrated the same favorable effects of reverting to healthier profiles--diet and physical activity--on breast cancer recurrences and prognosis [40]. Similar trends are observed in the Eastern Hemisphere, in Japan [21] and the need of an integrated approach is warranted [41]. Even in our country, Italy, Adherence to nutritional guidelines, and, notably, to Mediterranean diet, shows a continuous decline in the past 15 years (Figure 7).

This is the reason that in several countries, including USA, Mediterranean diet that encompasses advices for physical activity and moderate alcohol (wine) habits, is proposed as a simplified but strong paradigm of healthy lifestyle, coincident with the contemporary guidelines [42-44], strategically useful for promoting PPPM. In this dietary context, the use of coffee is of benefit for arterial hypertension through some renal mechanism [45,46] and for fatty liver through the increase of insulin sensitivity [47]. Multiple lifestyle behaviors have effects on health risks like obesity and other health outcomes. In order to develop effective primary prevention strategies, it would be important to consider multiple health indices when identifying high risk groups [48].

## The cluster of clinical skills

The opportunity of a more widespread cluster of clinical skills inside small group of clinical practice is usually recognized, but not demonstrated and even studied, and is currently considered a strategy timely and responsive to the needs of patients. It should be a shortcut toward effectiveness and a sustainable cost-benefit balance, in the era of healthcare cost restraint. This approach should include guidelines and expert recommendations for di-agnostic assessment and follow-up activities. An enormous library of textbooks, manuals, scientific articles is, obviously, available. The need of a conceptualization of the essential issues that can drive the PPPM approach of any medical doctor is strongly and widely perceived.

Clinical nutrition and dietary intervention are neglected aspects of the clinical prescriptions. Knowledge and skills background on medical doctors and health professionals is limited and capability of explicit and protracted interventions are substantially inexistent.

Despite the efforts to improve quality of medical curricula, it is still necessary an enhancement of knowledge and training for undergraduate and postgraduates medical students. This intervention will benefit from mandatory CME course for medical doctors and professionals (including psychologists) and the assessment of the outcome (change of clinical approach with patients). In health systems mainly funded by the States (public) this intervention is possible and suitable of analysis of the outcomes, in terms of benefit for several health indexes and of pharmaceutical and diagnostic procedures expenses.

**Figure 8:**
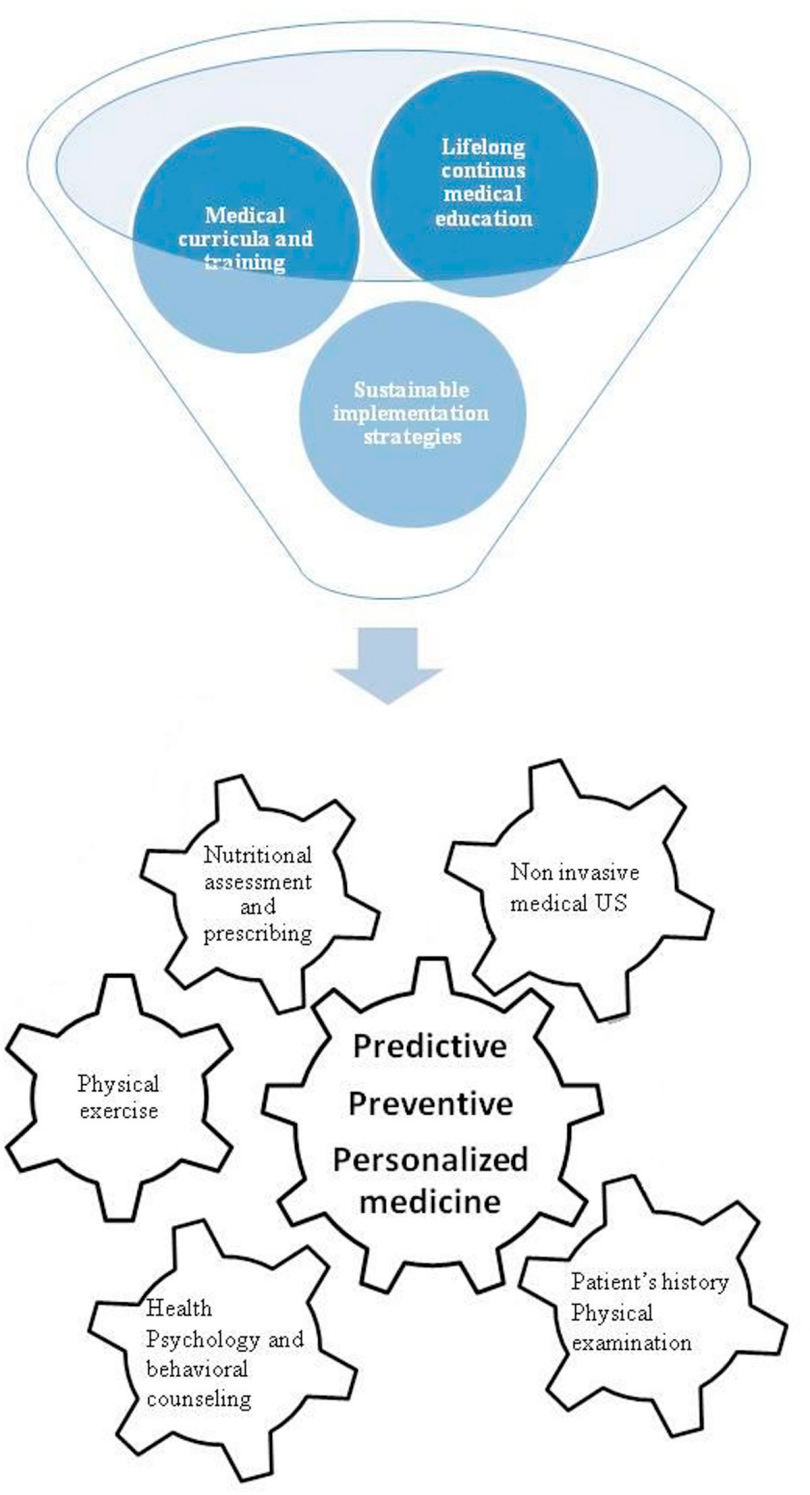
**Cluster of skills and knowledge for boosting a sustainable and effective PPPM**. Knowledge and skills in health psychology, nutritional and physical activity assessment and prescribing can be enhanced through appropriate funding of Continuous Medical Education (CME), also by professionally targeted e-learning. Professional post-graduate training and facilitation for purchasing friendly and affordable equipment facilities, mostly along a professionally driven expertise in US non-invasive procedures, is the other fundamental tool for enhancing an effective bedside PPPM.

Nutritional guidelines and risk factor assessment skills and awareness are the necessary premise for a predictive approach to tailored prevention and personalized medicine (the clinical perspective of medical doctors, alone or in small associated teams). This mechanism can work and is largely sustainable and affordable for European Health and university systems (Figure 8). A renewed and greater appreciation of the traditional medical skills (medical history and physical examination) focused to cardiovascular and respiratory disease, cancer, liver and GI disease, disability (neurological, psychiatric, orthopedic & rheumatic disease) with a timely extension of the diagnostic skills with the current friendly and affordable non-invasive procedures will succeed if an appropriate cultural milieu, also through media actions, will be build-up and maintained. The easy identification and scoring of fatty liver and of liver fibrosis is the single direct morphological clue to the identification of a metabolic-nutritional disease (even in the absence of obesity) and of consistent cardiovascular and renal risks [49-51]. The meaning and the power of laboratory information cannot be disregarded: nonetheless, different and recent approaches are putting into evidence new and unexpected biomarkers of obesity, that, conceivably, are also infectious causative factors [52,53]. The integration of resolute intervention on lifestyles (nutrition, exercise, habits) by psychological behavioral approach with expert and sustainable instrumental tools of assessment and follow-up give a substantial and cost-benefit valuable contribution. This is particularly important for diseases and conditions in which most research and effort are in the domain of pharmacology and multi-specialist diagnostic assessment and follow-up [54,55].

The background and current state of the knowledge warrant strongly a wider use at the bedside, in emergency and in the general practice of medical ultrasound diagnostics, abdominal, cardiac, vascular and of the thyroid-neck area. This is a true evidence-based challenge against the excessive expenses deriving from delay and uncertainty in diagnostics, involving often different specialists and in different places, with deplorable waiting lists.

The expert recommendations to solve the problem include dissemination, educational and research strategies and, principally, to build-up clinical excellence of younger doctors. The cluster of professional and expert skills, at the individual and group level is focused to predictive medicine (nutritional and physical activity assessment) and a friendly imaging of the most common and also rare disease. The preventive actions will include interventions on nutrition-friendly schools (according to food and nutrition action plans in the European Region and to the nutrition policy database for the WHO European Region) aimed at preventing micronutrient deficiencies and at the childhood obesity surveillance, monitoring the progress of improvements in nutrition, physical activity and reduction of obesity. Timely information on the most likely associated conditions (fatty liver and thyroid disease) is possible if multi-expertise of the single medical doctor is available. Tailored, personalized medicine is possible if it is easily available a tailored and timely diagnosis and nutritional profile assessment. This will allow affordable strategies for a widespread appropriate medical prescribing of nutrition and lifestyle change, with friendly and sustainable monitoring strategies. The place of a behavioral health psychology approach is central in this strategy. The assessment of illness perception and the enhancement of motivation and self-efficacy, some of the psychological markers of compliance and likely success of prescriptions, are the necessary premises for reaching adherence to lifestyle changes [56-58]. The role of the dietitian and of the physical activity counselor and instructor must share skills and competence of psychological feature: equally, medical doctors can obtain adequate compliance to medical prescription by appropriate approaches.

## Conclusion

### Outlook

Implementation of widespread friendly and non-invasive diagnostic procedures will be effective and reasonably with a favorable cost-benefit ratio if a consistent and diffuse professional enhancement of skills in health psychology, nutritional and physical activity assessment and prescribing will be available. A reference paradigm of a PPPM approach is possible inside a cultural framework of competences and skills in which the Medical Doctors can personally manage the need of prediction (early diagnosis), prevention (intervention on healthy persons) and tailored therapy and follow-up for patients. Overall this profile, flexible and adjustable according to specific needs and preferences due to different economic and ethno-cultural milieus, can be warranted as an operative paradigm.

### Recommendation

Knowledge and skills in health psychology, nutritional and physical activity assessment and prescribing can be enhanced through appropriate funding of Continuous Medical Education (CME), also by professionally targeted e-learning. Professional post-graduate training and facilitation for purchasing friendly and affordable equipment facilities, mostly along a professionally driven expertise in US non-invasive procedures, is the other fundamental tool for enhancing an effective bedside PPPM.
